# Effects of Digital Interventions on Symptom Improvement and Quality of Life in Patients With Overactive Bladder: Systematic Review and Meta-Analysis

**DOI:** 10.2196/88971

**Published:** 2026-08-10

**Authors:** Ni Wang, Fan Fan, Yuan Ou, Shanhe Huang, Chigang Cao, Hai Huang, Hao Huang

**Affiliations:** 1 Sun Yat-sen Memorial Hospital Guangzhou, Guangdong China

**Keywords:** overactive bladder, urinary incontinence, digital health, telemedicine, mobile apps, telerehabilitation, self-management, systematic review, meta-analysis

## Abstract

**Background:**

Overactive bladder (OAB) is a prevalent condition that substantially impairs quality of life (QoL); however, the real-world utility of standard behavioral and pharmacological therapies is often limited by poor long-term adherence. Digital interventions have emerged as a promising strategy to provide accessible and personalized support, but their overall efficacy compared with conventional care remains to be systematically established.

**Objective:**

This study aimed to evaluate the efficacy of digital interventions compared with conventional care for improving symptoms, QoL, and treatment adherence in patients with OAB.

**Methods:**

We conducted a systematic review and meta-analysis using data from PubMed, Embase, the Cochrane Library, and other databases from inception to July 2025. Randomized controlled trials (RCTs) assessing technology-based digital interventions for adult patients with OAB were included. The primary outcomes were objective symptom improvement (measured by bladder diaries) and subjective symptom scores, while secondary outcomes included QoL and attrition rates. Data were pooled using a random-effects model, and subgroup analyses were performed according to the delivery and supervision category (automated or app-based therapeutic content, telemedicine-supported care management, or digitally supervised behavioral or rehabilitation support) and intervention duration.

**Results:**

Seven RCTs involving 544 participants were included. Digital interventions were associated with a significant improvement in overall objective symptom indicators compared with conventional care (mean difference [MD] −1.72, 95% CI −2.61 to −0.83; *P*<.001), specifically reducing 24-hour urinary frequency (MD −2.55, 95% CI −4.22 to −0.89; *P*=.003) and urge incontinence episodes (MD −2.07, 95% CI −2.72 to −1.42; *P*<.001). The pooled analysis of subjective symptom scores did not reach statistical significance (standardized MD [SMD] −1.07, 95% CI −2.18 to 0.05; *P*=.06). Exploratory subgroup analysis suggested that digitally supervised behavioral or rehabilitation support had the largest point estimate (SMD −2.51, 95% CI −5.03 to 0.01), although the CI crossed the line of no effect, and the finding was based on 2 heterogeneous studies. The prespecified subgroup analysis by intervention duration showed that interventions lasting 6 to 13 weeks were associated with symptom reduction (SMD −1.41, 95% CI −2.70 to −0.11; *P*=.03), suggesting a potentially favorable duration range. Furthermore, digital interventions significantly improved QoL (SMD 1.47, 95% CI 0.11-2.84; *P*=.03). No significant difference was observed in dropout rates between digital and conventional care groups (risk ratio 0.92, 95% CI 0.59 to 1.45; *P*=.72).

**Conclusions:**

Digital interventions may improve objective symptoms and QoL in patients with OAB, with comparable short-term attrition to conventional care. Interventions with higher-intensity digital supervision may have potential for symptom reduction, but this finding remains uncertain because of the small number of heterogeneous studies. These findings may inform future stepped care models, but further well-designed, longer-term RCTs are needed to confirm optimal implementation strategies.

**Trial Registration:**

PROSPERO CRD420251164488; https://www.crd.york.ac.uk/PROSPERO/view/CRD420251164488

## Introduction

Overactive bladder (OAB) is a prevalent and burdensome clinical syndrome characterized by urinary urgency with or without urgency incontinence, often accompanied by frequency and nocturia [1]. Affecting an estimated 360 million individuals worldwide in 2020, with projections exceeding 400 million by 2030, OAB is associated with substantially impaired health-related quality of life (QoL) and an increased burden of psychosocial comorbidities such as depression and anxiety [2-7]. While effective first-line behavioral and pharmacological therapies are available, their real-world effectiveness is often limited by a critical challenge: poor long-term adherence [8,9].

Patient adherence data starkly illustrate the limitations of current treatment models. For example, adherence to behavioral therapies, a cornerstone of OAB management, can decline to as low as 32% after 1 year. Similarly, persistence with oral medications is suboptimal, with rates ranging from 15% to 40%, and annual discontinuation rates for some agents reaching as high as 85% [10-12]. This discrepancy between the established efficacy of treatments and their real-world effectiveness highlights a critical unmet need for interventions that can sustain long-term patient engagement and improve self-management skills.

Digital interventions—technology-based interventions that support remote diagnosis and management—have emerged as a promising strategy to overcome these barriers by providing accessible, scalable, and personalized support [13-15]. By offering accessible education, behavioral coaching, and symptom tracking, these interventions are designed to empower patients and reinforce their treatment plans [16-19]. Such interventions have demonstrated effectiveness in improving symptom control and QoL in other chronic conditions [20-24]. Applying this model to OAB is a logical extension, and preliminary studies have shown promise, with some reporting high rates of symptom improvement and patient satisfaction [25,26]. However, the existing evidence base remains fragmented. These early-phase trials are often limited by small sample sizes, heterogeneous intervention designs, and a lack of standardized outcome measures, making it difficult for clinicians to ascertain the overall magnitude of effect or identify optimal implementation strategies [27-29]. Consequently, the role of digital interventions in the broader OAB care paradigm remains uncertain.

Given the potential of digital health to transform OAB management and the need for a comprehensive evidence synthesis, we conducted a systematic review and meta-analysis of randomized controlled trials (RCTs). The objective was to evaluate the effectiveness of digital interventions on symptom improvement, QoL, and treatment adherence in patients with OAB.

## Methods

### Study Design and Protocol

This systematic review and meta-analysis followed the PRISMA (Preferred Reporting Items for Systematic Reviews and Meta-Analyses) reporting guideline [30]. The protocol was registered with PROSPERO (CRD420251164488).

### Eligibility Criteria

We included RCTs enrolling adult patients with nonneurogenic OAB. Eligible interventions were technology-enabled interventions delivered fully or partially through websites, mobile apps, telemedicine platforms, videoconferencing, telephone, WeChat, or other digital communication tools to support OAB education, self-management, monitoring, follow-up, behavioral training, or rehabilitation. This definition was informed by the World Health Organization (WHO) classification of digital health interventions and the digital therapeutics and mobile health (mHealth) literature [31-33]. The comparator was any control condition, including standard therapeutic regimens or usual care. The primary outcome was the severity of OAB symptoms, measured by objective data from bladder diaries or subjective symptom scores. Secondary outcomes included QoL and attrition rates. Studies were excluded if they were not original research, were not published in English or Chinese, or had no full text available.

### Information Sources and Search Strategy

Electronic databases, including PubMed, Embase, the Cochrane Library, CINAHL, Web of Science, China National Knowledge Infrastructure, Wanfang, and SinoMed, were searched from inception to July 2025. Search terms included a combination of controlled vocabulary (eg, “Urinary Bladder, Overactive”) and free text (eg, “telemedicine” and “mobile health”). A “snowball” search of relevant articles was also performed. The whole search strategy for all databases is presented in Multimedia Appendix 1.

### Study Selection and Data Extraction

After duplicate removal, 2 reviewers independently screened titles and abstracts using the ASReview LAB, a web-based machine learning application developed by Utrecht University, to enhance efficiency. Full texts of potentially eligible articles were then assessed against predefined inclusion criteria. Disagreements were resolved through discussion or by a third reviewer. Data were extracted by 1 reviewer using a standardized template and cross-checked by a second reviewer.

### Risk-of-Bias Assessment

The methodological quality of included RCTs was appraised using the Cochrane risk of bias 2 tool [34]. Two reviewers independently performed the assessment, evaluating 5 domains: the randomization process, deviations from intended interventions, missing outcome data, measurement of the outcome, and selection of the reported results.

### Data Analysis

To prepare the data for synthesis, the necessary conversions were performed. For studies that reported SE instead of SD, the SD was calculated using the formula SD=SE×√N, where N is the sample size. For studies reporting the median and IQR, we estimated the mean and SD using the method described by Wan et al [35]. Meta-analyses were performed using Review Manager (RevMan), version 5.3 (The Cochrane Collaboration). For continuous outcomes reported on different scales, standardized mean differences (SMDs) with 95% CIs were calculated. For outcomes measured on the same scale, mean differences (MDs) were used to compare the results. Statistical heterogeneity was assessed using the *I*² statistic. Pooled estimates were generated using a random-effects model. To explore potential sources of heterogeneity, we performed prespecified subgroup analyses based on intervention duration and the delivery and supervision category. Specifically, interventions were classified into three categories based on their primary therapeutic function and intensity of digital supervision

First, automated or app-based therapeutic content, including self-guided or semiautomated digital programs delivering structured education; behavioral strategies; symptom tracking; or self-management support through websites, software modules, or avatars.

Second, telemedicine-supported care management, including interventions using video visits, telephone follow-up, or audiovisual communication primarily to replace or supplement routine clinical follow-up and care pathway management.

Third, digitally supervised behavioral or rehabilitation support, including interventions in which digital tools were used to provide active professional support for behavioral training, rehabilitation, or treatment adherence. This category was defined by the intensity and function of digital support rather than by identical therapeutic content. Examples included synchronous video-supervised exercise and frequent remote adherence reinforcement through WeChat-based check-ins and telephone follow-up.

Telemedicine-supported care management focused primarily on replacing or supplementing routine clinical follow-up, whereas digitally supervised behavioral or rehabilitation support involved a more active role in reinforcing behavioral training, rehabilitation activities, or treatment adherence. Because interventions within the latter category differed in therapeutic content, findings from this subgroup were interpreted as exploratory.

Given that fewer than 10 trials were included, a formal assessment of publication bias was not conducted.

### Certainty of Evidence Assessment

The certainty of the evidence for each outcome was evaluated using the GRADE (Grading of Recommendations Assessment, Development and Evaluation) framework [36]. The appraisal was based on 5 domains: risk of bias, inconsistency, indirectness, imprecision, and publication bias.

## Results

### Search Results and Study Characteristics

**Figure 1:**
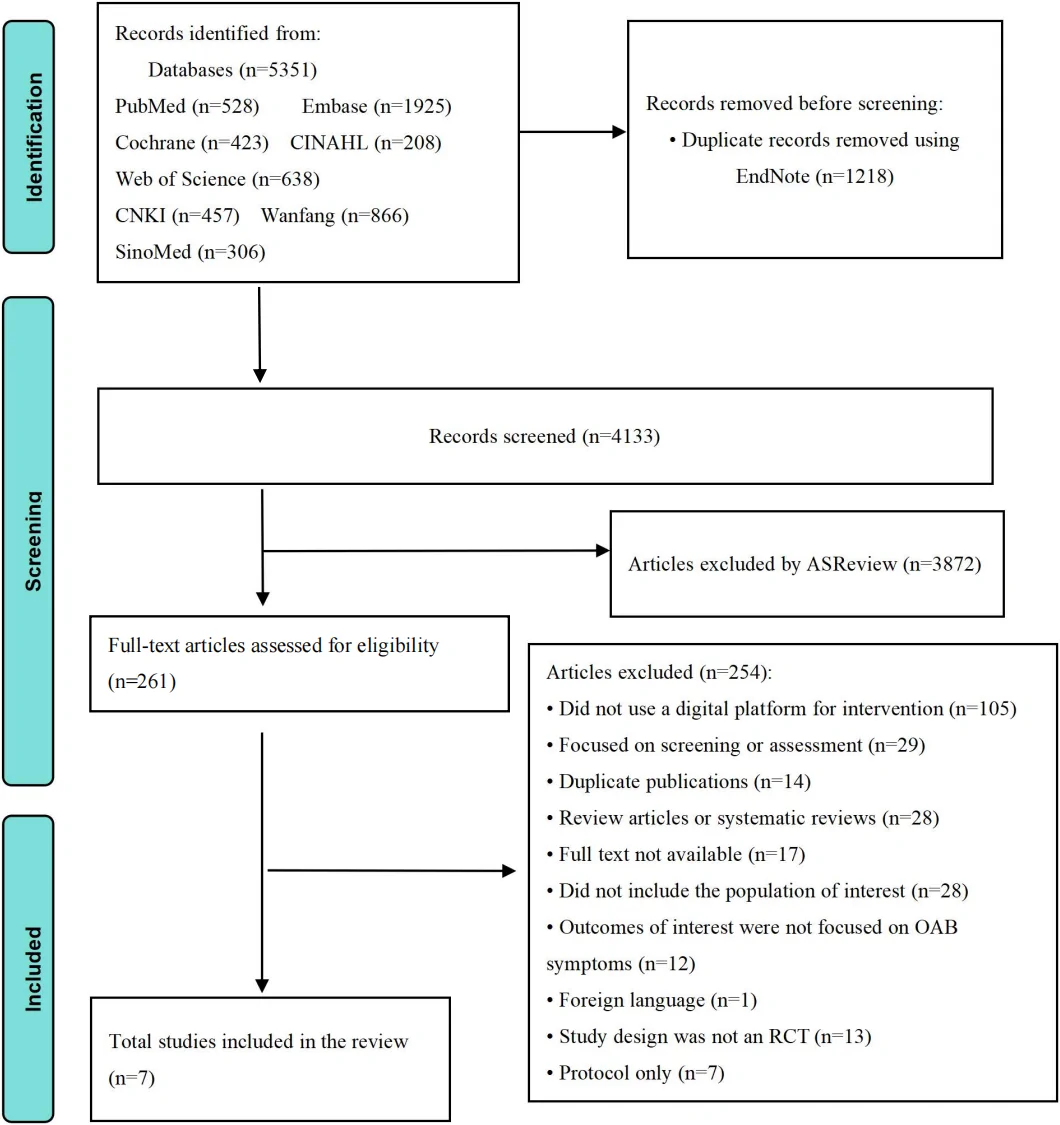
Study retrieval and selection flowchart (PRISMA [Preferred Reporting Items for Systematic Reviews and Meta-Analyses]). OAB: overactive bladder; RCT: randomized controlled trial.

The literature search yielded 5351 records. After duplicate removal, 4133 titles and abstracts were screened, leading to the assessment of 261 full-text articles for eligibility. Ultimately, 7 RCTs met the inclusion criteria (Figure 1). The studies were conducted in the United States (n=4, 57%), Egypt (n=1, 14%), China (n=1, 14%), and Japan (n=1, 14%).

The study populations consisted predominantly of female participants, reflecting the epidemiology of OAB, although 2 trials [16,19] also included male participants. The sample sizes ranged from 36 to 164 participants, with mean ages ranging from 39.0 years [17] to 63.5 years [18]. The duration of interventions ranged from 12 weeks to 1 year, with most studies lasting 12 weeks. Detailed characteristics of the included studies are summarized in Multimedia Appendix 2. The certainty of evidence for each outcome is summarized in Multimedia Appendix 3. Reasons for exclusion of full-text reports are summarized in Multimedia Appendix 4. The digital interventions varied significantly in delivery mode and intensity. On the basis of our prespecified criteria, the included studies were categorized into 3 categories.

First, automated or app-based therapeutic content (2/7, 28%): this category included the study by Andrade et al [37], which used an online program featuring virtual avatars, and the study by Funada et al [18], which delivered a structured multicomponent program that included self-monitoring, education, cognitive behavioral therapy–based behavioral strategies, lifestyle modification, pelvic floor muscle training (PFMT), and bladder training. Both interventions relied mainly on structured therapeutic content and patient self-management.

Second, telemedicine-supported care management (3/7, 43%): this group included studies that used video visits [16], audiovisual calls [19], or telephone follow-ups [38] to replace or augment standard clinical visits and OAB care pathway management.

Third, digitally supervised behavioral or rehabilitation support (2/7, 28%): Alghitany et al [17] delivered a 12-week synchronous video-supervised Pilates program led by a physiotherapist. Li et al [39] delivered a Predisposing, Reinforcing, and Enabling Constructs in Educational Diagnosis and Evaluation–Policy, Regulatory, and Organizational Constructs in Educational and Environmental Development (PRECEDE-PROCEED) model–based behavioral self-management and health education intervention that included structured education sessions, individualized guidance on lifestyle management, urge suppression techniques, PFMT, and bladder training, as well as WeChat group support, daily adherence checks, and weekly telephone follow-up. These interventions shared active professional digital support but differed substantially in their therapeutic content and comparator conditions.

Control conditions varied across trials and included routine outpatient follow-up, standard health education, wait-list control, a voice-only version of the online program, and an unsupervised home Pilates program.

### Risk of Bias in Included Studies

**Figure 2:**
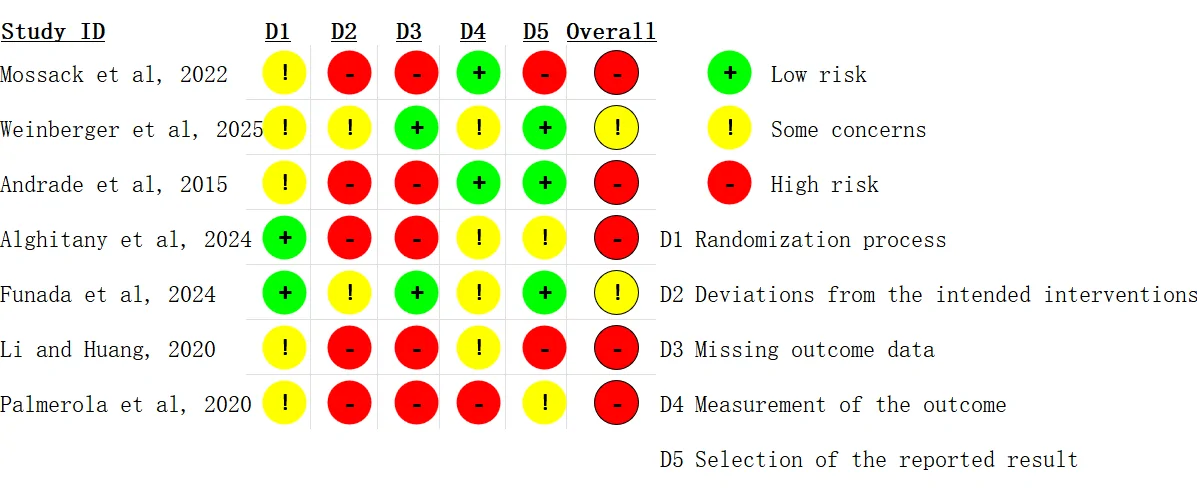
Results of quality appraisal.

The risk-of-bias assessment for each included study is presented in Figure 2. Five trials raised “some concerns” regarding the randomization process due to insufficient reporting of allocation concealment. In the domain of deviations from intended interventions, 5 trials were judged to be at high risk of bias, and 2 trials raised some concerns, mainly because blinding of participants and personnel was not feasible in behavioral and digital intervention trials. Five trials were also judged to be at high risk of bias due to missing outcome data. Overall, 5 trials were rated as having high risk of bias and 2 as having some concerns. These methodological limitations may have affected the reliability of the pooled estimates and should be considered when interpreting the findings.

### Effects of Interventions

The main findings are presented in the subsequent sections.

#### Effects on Objective Symptom Indicators

**Figure 3:**
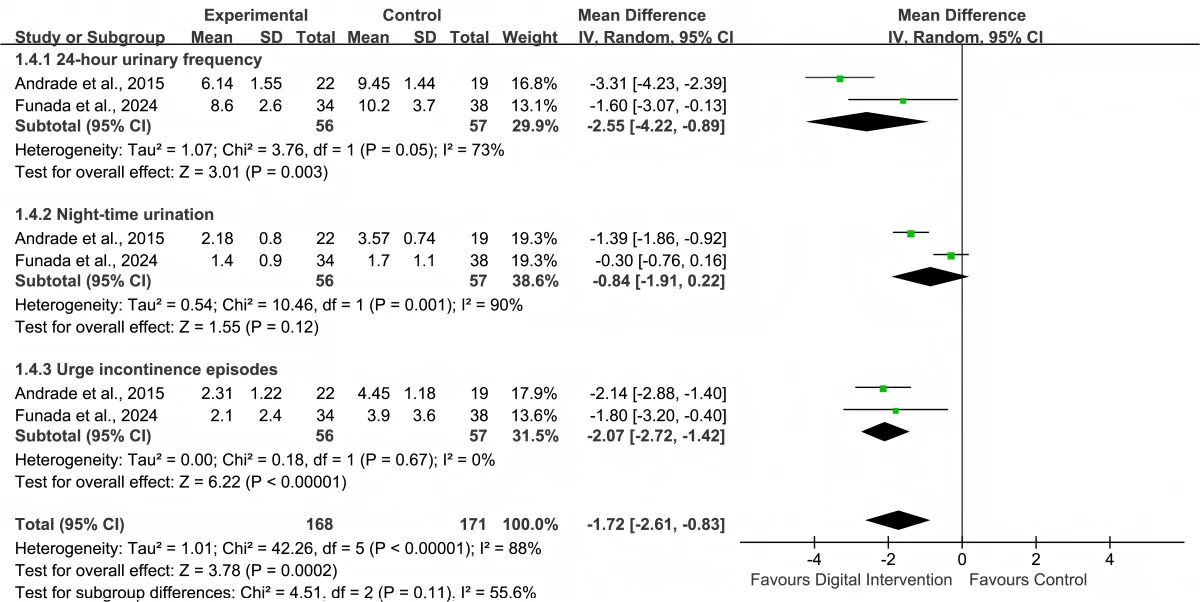
Forest plot of the objective symptom indicators.

Data from 2 RCTs [18,37] including 113 participants (56 in the digital intervention group and 57 in the control group) assessing objective symptoms via bladder diaries were pooled. Compared with conventional care, digital interventions were associated with a significant improvement in the overall objective symptom indicators (MD −1.72, 95% CI −2.61 to −0.83; *P*<.001; Figure 3). Specifically, significant reductions were observed in 24-hour urinary frequency (MD −2.55, 95% CI −4.22 to −0.89; Figure 3) and urge incontinence episodes (MD −2.07, 95% CI −2.72 to −1.42; *P*<.001; Figure 3). Notably, the analysis for urge incontinence showed zero heterogeneity (*I*^2^=0%), indicating highly consistent efficacy across studies. In contrast, no significant difference was observed for nighttime urination (MD −0.84, 95% CI −1.91 to 0.22; Figure 3), which exhibited substantial heterogeneity (*I*²=90%). This heterogeneity may be related to differences in intervention content, comparator design, baseline nocturia severity, and outcome assessment between the 2 contributing studies. In addition, nocturia is a multifactorial symptom that may be influenced by sleep disturbance, nocturnal polyuria, fluid intake, and comorbid conditions, which may not respond uniformly to OAB-focused digital interventions. As only 2 studies contributed data to this outcome, further subgroup or metaregression analyses were not feasible. The certainty of evidence was rated as moderate for urinary frequency and urge incontinence but very low for nighttime urination (Multimedia Appendix 3).

#### Effects on OAB Symptom–Specific Questionnaire Score

**Figure 4:**
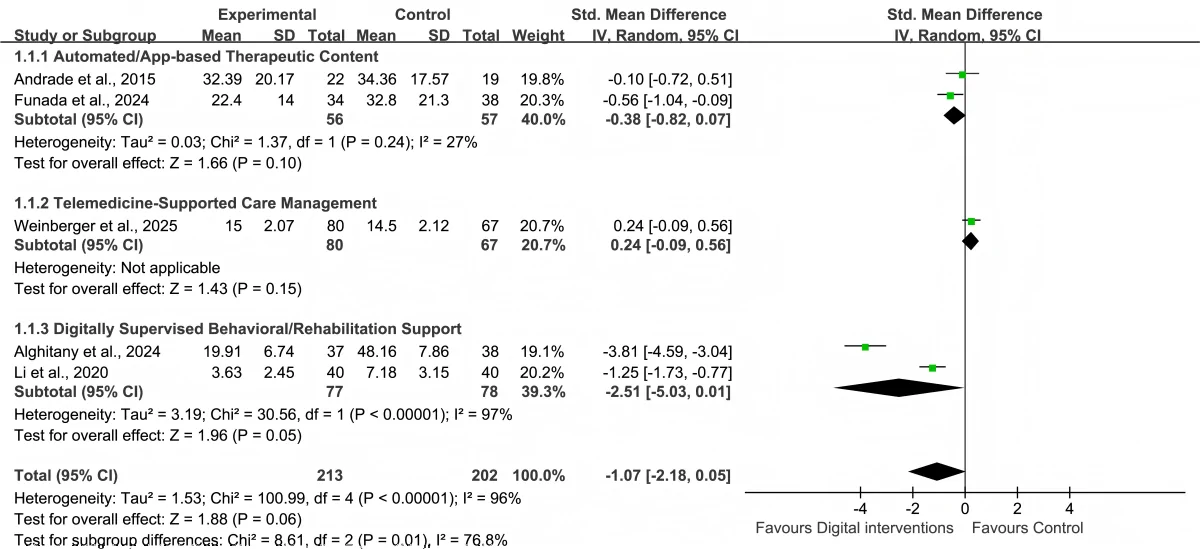
Forest plot of the overactive bladder symptom–specific questionnaire score: subgroup analysis by intervention type.

**Figure 5:**
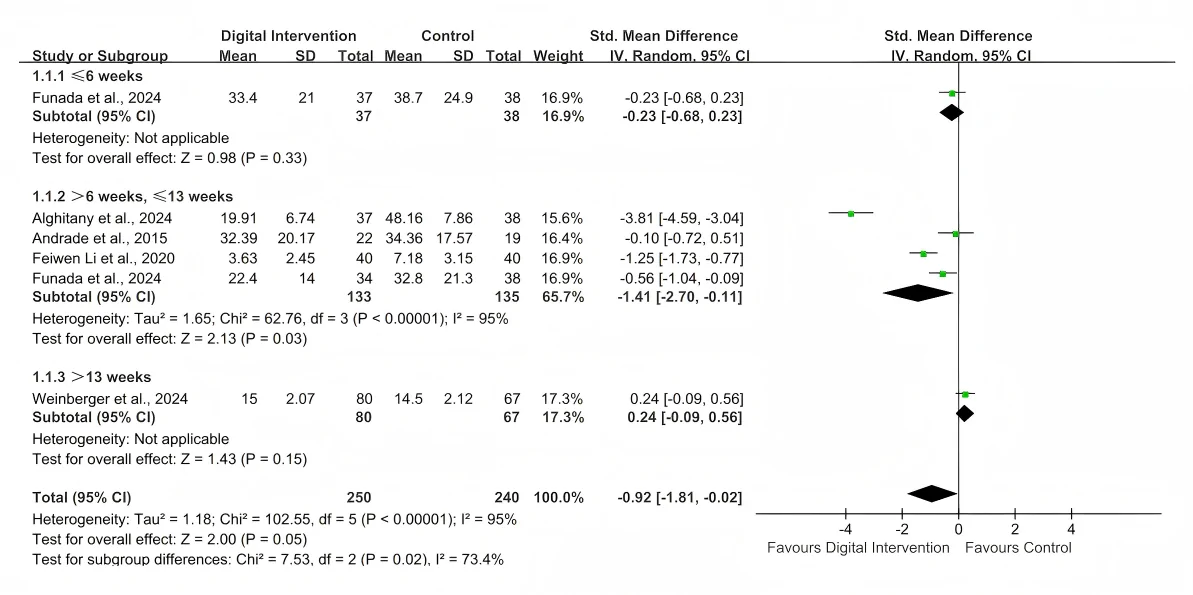
Forest plot of the overactive bladder symptom–specific questionnaire score: subgroup analysis by intervention duration.

Five RCTs [16-18,37,39] including 415 participants provided data on subjective symptom scores. The pooled analysis showed no statistically significant difference in subjective symptom scores (SMD −1.07, 95% CI −2.18 to 0.05; *P*=.06; Figure 4), with substantial statistical heterogeneity. To investigate the source of heterogeneity, a subgroup analysis based on intervention delivery mode was performed, revealing significant differences between subgroups (*P*=.01; Figure 4). Specifically, digitally supervised behavioral or rehabilitation support showed the largest point estimate (SMD −2.51, 95% CI −5.03 to 0.01; Figure 4). However, this exploratory subgroup was defined by the intensity and function of digital professional support rather than by identical therapeutic content. The CI crossed the line of no effect, and substantial within-subgroup heterogeneity was observed (*I*²=97%). Therefore, this subgroup finding should be interpreted cautiously. Although both individual studies showed statistically significant effects favoring the digital intervention, the pooled random-effects estimate was not statistically significant because the studies differed substantially in intervention content, comparator conditions, and effect magnitude. By contrast, automated or app-based therapeutic content showed a smaller effect estimate favoring digital therapy that was not statistically significant (SMD −0.38, 95% CI −0.82 to 0.07; *P*=.10; Figure 4), with low within-subgroup heterogeneity (*I*²=27%; Figure 4). Telemedicine-supported care management showed an effect estimate close to zero (SMD 0.24, 95% CI −0.09 to 0.56; Figure 4). Furthermore, the prespecified subgroup analysis based on intervention duration showed that interventions lasting 6 to 13 weeks were associated with improvement in OAB symptoms (SMD −1.41, 95% CI −2.70 to −0.11; *P*=.03; Figure 5). The certainty of evidence for this outcome was very low due to a serious risk of bias and severe inconsistency, although the subgroup analysis by delivery mode offers a plausible explanation for the observed heterogeneity (Multimedia Appendix 3).

#### Effects on QoL

**Figure 6:**
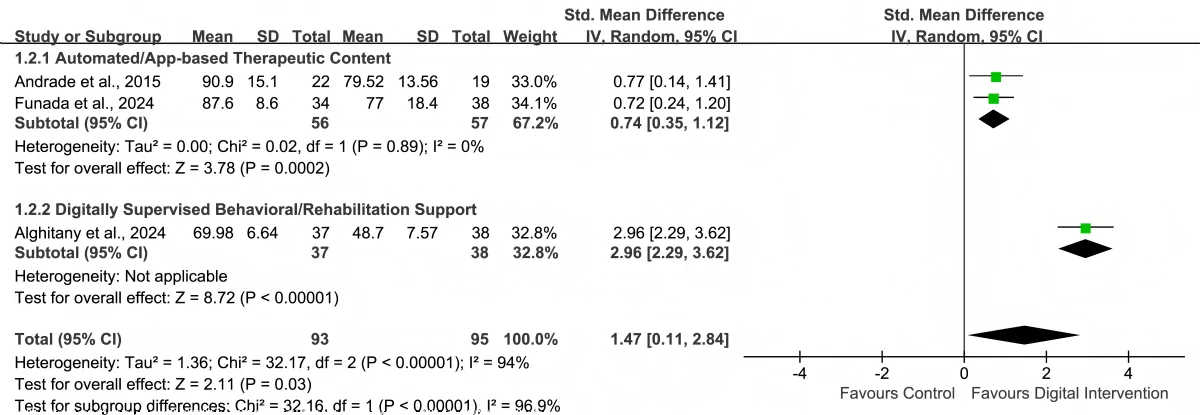
Forest plot of the overactive bladder–specific quality of life score: subgroup analysis by intervention type.

Three RCTs [17,18,37] including 188 participants reported on OAB-specific QoL. The pooled analysis demonstrated a significant improvement in QoL (SMD 1.47, 95% CI 0.11-2.84; *P*=.03; Figure 6). However, substantial heterogeneity was observed (*I*^2^=94%; Figure 6). A subgroup analysis based on the delivery and supervision category explained part of this heterogeneity (*P*<.001 for subgroup differences). Digitally supervised behavioral or rehabilitation support was associated with a large effect estimate for QoL improvement (SMD 2.96, 95% CI 2.29-3.62; Figure 6). However, this estimate was derived from a single study of video-supervised Pilates and therefore should not be interpreted as evidence for the broader digitally supervised behavioral or rehabilitation support category. In contrast, automated or app-based therapeutic content showed a moderate but consistent benefit (SMD 0.74, 95% CI 0.35-1.12; Figure 6), with no heterogeneity within this subgroup (*I*^2^=0%). The certainty of evidence was initially rated as low due to risk of bias and inconsistency (Multimedia Appendix 3). However, the subgroup analysis provides a plausible explanation for the observed heterogeneity.

#### Effects on Attrition Rates

**Figure 7:**
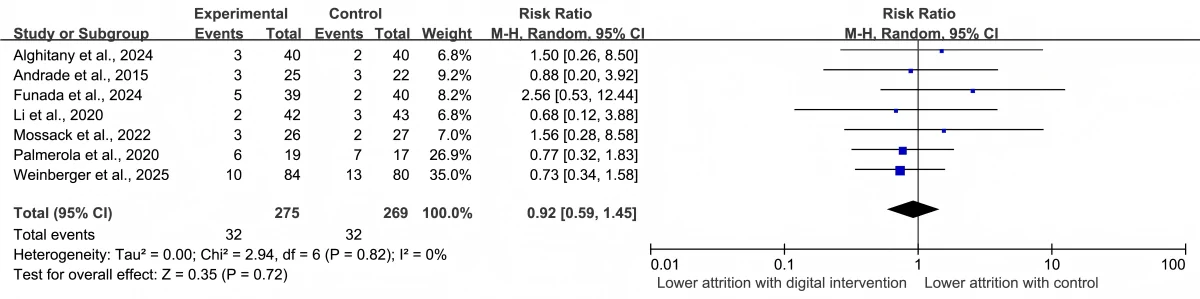
Forest plot of the effects of digital interventions on attrition rates.

Seven RCTs [16-19,37-39] including 544 participants reported attrition rates. The pooled analysis revealed no significant difference in the risk of dropout between the intervention and control groups (risk ratio 0.92, 95% CI 0.59-1.45; *P*=.72; Figure 7). The certainty of evidence for this outcome was low due to serious risk of bias and imprecision (Multimedia Appendix 3).

## Discussion

### Principal Findings

This systematic review and meta-analysis of 7 RCTs suggests that digital interventions may improve objective OAB symptoms—specifically urinary frequency and urge incontinence—and QoL compared with conventional care. A notable exploratory finding is that the observed heterogeneity in subjective outcomes may be partly related to intervention delivery mode and supervision intensity. Digitally supervised behavioral or rehabilitation support showed the largest point estimate; however, this exploratory finding was based on 2 studies with substantially different therapeutic content and high heterogeneity. Automated or app-based therapeutic content showed smaller and more consistent, although nonsignificant, effect estimates, whereas telemedicine-supported care management showed effects close to those of the control conditions. Our prespecified duration subgroup analysis suggested that interventions lasting 6 to 13 weeks were associated with subjective symptom improvement, indicating a potential therapeutic window for digital OAB interventions. No significant difference was observed in attrition rates between digital and conventional care groups, suggesting comparable short-term acceptability. These findings support further evaluation of digital interventions in OAB management and align with broader evidence on the use of mHealth for other chronic conditions [40-42].

### Impact of Delivery Mode and Supervision Intensity

The digitally supervised behavioral or rehabilitation support subgroup showed the largest point estimate for subjective symptom reduction, but this finding should be interpreted cautiously because the subgroup included only 2 studies, the pooled estimate was not statistically significant, and substantial heterogeneity was observed (*I*²=97%). This subgroup was defined by the intensity and function of digital support rather than by identical therapeutic content. Alghitany et al [17] delivered video-supervised Pilates training, whereas Li and Huang [39] delivered PRECEDE-PROCEED model–based health education with individualized PFMT and bladder training instruction, WeChat-based adherence reinforcement, and telephone follow-up. These differences in intervention content and comparator conditions may have contributed to the high heterogeneity. The finding should therefore be considered exploratory and should not be interpreted as evidence that a specific telerehabilitation modality is superior.

In contrast, automated or app-based therapeutic content showed a smaller and more consistent, although nonsignificant, effect estimate (*I*²=27%). In our classification, the significant improvement in objective measures, such as urinary frequency, was derived from this category. This aligns with broader evidence showing that core components of behavioral therapy are practical for OAB, and our findings suggest that digital platforms may be a viable delivery mechanism [43-46]. Such platforms, using embedded avatars or automated coaching, may provide scalable and accessible support without requiring continuous clinician availability [47].

### Clinical Positioning of Telemedicine

It is important to distinguish the clinical value of telemedicine-supported care management from that of other modalities. Although this subgroup did not show superior symptom reduction compared to control, this aligns with the noninferiority design of the included trials. The primary value of these interventions lies not in therapeutic intensification but in health care efficiency and accessibility. Interventions leveraging telemedicine offer the advantage of direct, albeit virtual, patient-clinician interaction, which may enhance personalized feedback and patient trust [48]. Our findings support a stratified approach: telemedicine may be more suitable for patients requiring complex guidance, whereas automated or app-based therapeutic content may be ideal for those who are technologically proficient and prefer self-directed care [49].

### Optimal Duration and Attrition

Our duration subgroup analysis suggested that interventions lasting 6 to 13 weeks may be associated with subjective symptom improvement. This finding may reflect a balance between early habit formation and potential digital health fatigue. Consistent with previous research on mHealth engagement [50], user interaction with digital tools often wanes after the initial novelty fades. Therefore, interventions lasting 6 to 13 weeks may provide a reasonable period for establishing initial behavioral patterns, such as bladder training habits, before the burden of daily logging leads to disengagement. However, this duration finding should be viewed as supportive rather than definitive because the exact duration cutoffs were derived from the distribution of included studies.

Interestingly, while our introduction highlighted poor long-term adherence as a key limitation of standard care, our analysis of attrition rates in a trial setting showed no significant advantage for digital interventions over control groups. This finding should not be interpreted as evidence that digital interventions improve retention compared with conventional care. However, it is also clinically relevant that digital interventions did not increase dropout rates, suggesting comparable short-term acceptability and alleviating concerns that technology-based interventions might impose an additional burden on patients [51,52]. As most included trials were relatively short and attrition is only a crude proxy for real-world engagement, whether digital interventions can improve long-term adherence in OAB management remains uncertain. Combined with the duration finding, these results may inform future stepped care strategies in which higher-intensity digital support is used during a potential early treatment window, followed by lower-intensity maintenance support to prevent fatigue.

### Implications and Future Directions

These findings offer several practical implications. On the basis of the potential for stepped care, a hybrid model—combining an initial telemedicine consultation with app-based self-management—could be a promising future direction. Further economic and implementation studies are needed before reimbursement strategies for digital OAB interventions can be determined. For technology developers, the challenge lies in creating adaptive interventions, potentially using AI, that can be tailored to individual patient profiles and engagement patterns [53-55]. While our findings are promising, larger, more methodologically robust RCTs are necessary to validate these results. Crucially, trials comparing different digital modalities (eg, synchronous telemedicine vs asynchronous app-based coaching) are essential to guide personalized treatment selection.

### Limitations

First, our findings are based on a small number of included studies (n=7) and a limited total sample size, which may affect the robustness of the results, particularly for subgroup analyses. Second, several included trials had high or unclear risk of bias, particularly regarding deviations from intended interventions and missing outcome data. Third, substantial heterogeneity remained in some analyses, especially in the digitally supervised behavioral or rehabilitation support subgroup (*I*^2^=97%), likely because the included studies differed in therapeutic content, such as video-supervised Pilates training vs PRECEDE-PROCEED model–based health education with WeChat and telephone support. For nocturia, heterogeneity was also substantial, likely reflecting the multifactorial nature of nighttime urination and differences between the 2 contributing trials. Fourth, the multicomponent nature of the interventions makes it difficult to isolate the specific elements responsible for the observed benefits. Finally, because fewer than 10 studies were included, funnel plot asymmetry and Egger-type tests were not performed, as these methods are unreliable with a small number of studies. Although we used a broad search strategy across multiple English and Chinese databases and performed citation tracking to reduce the risk of missing relevant published studies, small-study effects and publication bias could not be excluded. Therefore, the pooled effect estimates may be inflated if studies with null or unfavorable findings remain unpublished.

### Conclusions

In this systematic review and meta-analysis of 7 RCTs, digital interventions may reduce urinary frequency and urge incontinence episodes and improve OAB-specific QoL compared with conventional care. However, confidence in these findings is limited by the small number of trials, substantial heterogeneity, high or unclear risk of bias, and low or very low certainty for several outcomes. Exploratory subgroup analyses suggested that higher-intensity digitally supervised behavioral or rehabilitation support may be associated with greater symptom improvement, but this finding remains uncertain because of the small number of heterogeneous studies. Interventions lasting 6 to 13 weeks may be associated with symptom improvement, but the current evidence is insufficient to define an optimal intervention duration. Larger, methodologically rigorous, and longer-term RCTs are needed to confirm these findings and clarify the effectiveness, adherence, and implementation strategies of digital interventions for OAB.

## Appendix

Multimedia Appendix 1 Index and keyword terms used in databases.

Multimedia Appendix 2 Characteristics of the included studies.

Multimedia Appendix 3 Summary of findings.

Multimedia Appendix 4 List of excluded studies and reasons for exclusion.

Multimedia Appendix 5 PRISMA 2020 checklist.

## Abbreviations

GRADE: Grading of Recommendations Assessment, Development and Evaluation
MD: mean difference
mHealth: mobile health
OAB: overactive bladder
PFMT: pelvic floor muscle training
PRECEDE-PROCEED: Predisposing, Reinforcing, and Enabling Constructs in Educational Diagnosis and Evaluation–Policy, Regulatory, and Organizational Constructs in Educational and Environmental Development
PRISMA: Preferred Reporting Items for Systematic Reviews and Meta-Analyses
QoL: quality of life
RCT: randomized controlled trial
SMD: standardized mean difference
WHO: World Health Organization

## Data Availability

The data extracted and used for the meta-analysis are available from the corresponding author on reasonable request.
