# Supplementary material for: Effects of Digital Interventions on Symptom Improvement and Quality of Life in Patients With Overactive Bladder: Systematic Review and Meta-Analysis
Source: J Med Internet Res. 2026 Aug 10;28:e88971. doi: 10.2196/88971 (PMC13504251; doi:10.2196/88971)
Supplement: Supplementary file 2 [file jmir_v28i1e88971_app1.docx]

Multimedia Appendix 1. Index and keyword terms used in databases.

| **Database** |  | **Index and keyword terms** |
| --- | --- | --- |
| PubMed | #1  #2 | "Urinary Bladder, Overactive"[MeSH Terms] OR "overactive bladder"[tiab] OR "overactive bladder syndrome"[tiab] OR "bladder overactive"[tiab] OR "overactive urinary bladder"[tiab] OR "bladder overactivity"[tiab] OR "OAB"[tiab] OR "idiopathic overactive bladder"[tiab]  OR "Detrusor Overactivity"[MeSH Terms] OR "detrusor overactivity"[tiab] OR "overactive detrusor"[tiab] OR "overactive detrusor function"[tiab] OR "detrusor function, overactive"[tiab] OR "detrusor overactive"[tiab] OR "detrusor overactivities"[tiab] OR "detrusor instability"[tiab] OR "idiopathic detrusor overactivity"[tiab]  OR "Urinary Incontinence, Urge"[MeSH Terms] OR "Urge Incontinence"[tiab] OR "Incontinence, Urge"[tiab] OR "UUI"[tiab] OR "Urinary Reflex Incontinence"[tiab] OR "Incontinence, Urinary Reflex"[tiab] OR "Urinary Urge Incontinence"[tiab] OR "urge-type urinary incontinence"[tiab] OR "urgency incontinence"[tiab] OR "Storage urinary symptoms"[tiab]  "Telemedicine"[MeSH Terms] OR "Mobile Applications"[MeSH Terms] OR "Wearable Electronic Devices"[MeSH Terms]  OR "eHealth"[tiab] OR "mHealth"[tiab] OR "Electronic Health"[tiab] OR "Digital Health"[tiab] OR "Digital Therapeutics"[tiab] OR "Mobile Health"[tiab] OR "Digital Medicine"[tiab] OR "Telehealth"[tiab] OR "Virtual Care"[tiab]  OR "Digital Intervention"[tiab] OR "Technology-based Intervention"[tiab] OR "Computer-assisted Therapy"[tiab] OR "Internet Intervention"[tiab] OR "Remote Intervention"[tiab] OR "Virtual Intervention"[tiab] OR "Online Intervention"[tiab] OR "Computerized Intervention"[tiab] OR "Mobile Phone Intervention"[tiab] OR "App-based Intervention"[tiab]  OR "Mobile App"[tiab] OR "Mobile Apps"[tiab] OR "App, Mobile"[tiab] OR "Apps, Mobile"[tiab] OR "Mobile Application"[tiab] OR "Smartphone App"[tiab] OR "App-based"[tiab] OR "Smartphone"[tiab] OR "Health Apps"[tiab] OR "Wearable Devices"[tiab] OR "Smart Devices"[tiab]  OR "Web-based"[tiab] OR "Internet-based"[tiab] OR "Online"[tiab] OR "Teleconferencing"[tiab] OR "Text Messaging"[tiab] OR "SMS Intervention"[tiab] OR "Remote Monitoring"[tiab]  OR ("self-management"[tiab] AND ("digital"[tiab] OR "online"[tiab]))  OR "wechat"[tiab] OR "Portable Electronic Apps"[tiab] OR "Portable Electronic Applications"[tiab] OR "Electronic Application, Portable"[tiab] OR "Portable Electronic Application"[tiab] OR "Portable Software Apps"[tiab] OR "software"[tiab] OR "Portable Software Applications"[tiab] OR "Application, Portable Software"[tiab] OR "Portable Software Application"[tiab]  OR "Device, Wearable Electronic"[tiab] OR "Electronic Device, Wearable"[tiab] OR "Wearable Electronic Device"[tiab] OR "Wearable Devices"[tiab] OR "Device, Wearable"[tiab] OR "Wearable Device"[tiab] OR "Wearable Technology"[tiab] OR "Technology, Wearable"[tiab] OR "Wearable Technologies"[tiab] OR "Electronic Skin"[tiab] OR "Skin, Electronic"[tiab] OR "Wearable Computer"[tiab] OR "Computer, Wearable"[tiab] OR "Wearable Computers"[tiab]  OR "behaviour therapy"[MeSH Terms] OR "behaviour therapy"[tiab] OR "behavioral therapy"[tiab] OR "cognitive therapy"[tiab] OR "cognitive behavioural therapy"[tiab] OR "cognitive behavioral therapy"[tiab] OR "CBT"[tiab] OR "ICBT"[tiab] OR "internet-based CBT"[tiab] OR "online CBT"[tiab] OR "web-based CBT"[tiab] OR "app-based CBT"[tiab] OR "computerized CBT"[tiab] OR "digital CBT"[tiab] OR "e-CBT"[tiab] OR "cognitive restructuring"[tiab] OR "cognitive remediation"[tiab]  #1 AND #2 （528 2025-07-08) |
| EMBASE | #1  #2 | 'overactive bladder'/exp OR 'urinary Bladder, overactive':ti,ab,kw OR 'overactive bladder syndrome':ti,ab,kw OR 'bladder overactive':ti,ab,kw OR 'overactive urinary bladder':ti,ab,kw OR 'bladder overactivity':ti,ab,kw OR 'OAB':ti,ab,kw OR 'idiopathic overactive bladder':ti,ab,kw  OR 'detrusor overactivity'/exp OR 'detrusor overactivity':ti,ab,kw OR 'overactive detrusor':ti,ab,kw OR 'overactive detrusor function':ti,ab,kw OR 'detrusor function, overactive':ti,ab,kw OR 'detrusor overactive':ti,ab,kw OR 'detrusor overactivities':ti,ab,kw OR 'detrusor instability':ti,ab,kw OR 'idiopathic detrusor overactivity':ti,ab,kw  OR 'urge incontinence'/exp OR 'urge incontinence':ti,ab,kw OR 'incontinence, urge':ti,ab,kw OR 'UUI':ti,ab,kw OR 'urinary reflex incontinence':ti,ab,kw OR 'incontinence, urinary reflex':ti,ab,kw OR 'urinary urge incontinence':ti,ab,kw OR 'urge-type urinary incontinence':ti,ab,kw OR 'urgency incontinence':ti,ab,kw OR 'storage urinary symptoms':ti,ab,kw  ('telemedicine'/exp OR 'computer assisted therapy'/exp OR 'mobile application'/exp OR 'telehealth'/exp OR 'web-based intervention'/exp OR 'teleconference'/exp OR 'digital health'/exp OR 'wearable electronic device'/exp OR 'behaviour therapy'/exp)  OR  ('ehealth':ti,ab,kw OR 'mhealth':ti,ab,kw OR 'electronic health':ti,ab,kw OR 'digital health':ti,ab,kw OR 'digital therapeutics':ti,ab,kw OR 'mobile health':ti,ab,kw OR 'digital medicine':ti,ab,kw OR 'telehealth':ti,ab,kw OR 'virtual care':ti,ab,kw)  OR  ('digital intervention':ti,ab,kw OR 'technology-based intervention':ti,ab,kw OR 'computer-assisted therapy':ti,ab,kw OR 'internet intervention':ti,ab,kw OR 'remote intervention':ti,ab,kw OR 'virtual intervention':ti,ab,kw OR 'online intervention':ti,ab,kw OR 'computerized intervention':ti,ab,kw OR 'mobile phone intervention':ti,ab,kw OR 'app-based intervention':ti,ab,kw)  OR  ('mobile app':ti,ab,kw OR 'mobile apps':ti,ab,kw OR 'app, mobile':ti,ab,kw OR 'apps, mobile':ti,ab,kw OR 'mobile application':ti,ab,kw OR 'smartphone app':ti,ab,kw OR 'app-based':ti,ab,kw OR 'smartphone':ti,ab,kw OR 'health apps':ti,ab,kw OR 'wearable devices':ti,ab,kw OR 'smart devices':ti,ab,kw)  OR  ('web-based':ti,ab,kw OR 'internet-based':ti,ab,kw OR 'online':ti,ab,kw OR 'teleconferencing':ti,ab,kw OR 'text messaging':ti,ab,kw OR 'sms intervention':ti,ab,kw OR 'remote monitoring':ti,ab,kw)  OR  ('self-management':ti,ab,kw AND ('digital':ti,ab,kw OR 'online':ti,ab,kw))  OR  ('wechat':ti,ab,kw OR 'portable electronic apps':ti,ab,kw OR 'portable electronic applications':ti,ab,kw OR 'electronic application, portable':ti,ab,kw OR 'portable electronic application':ti,ab,kw OR 'portable software apps':ti,ab,kw OR 'software':ti,ab,kw OR 'portable software applications':ti,ab,kw OR 'application, portable software':ti,ab,kw OR 'portable software application':ti,ab,kw)  OR  ('device, wearable electronic':ti,ab,kw OR 'electronic device, wearable':ti,ab,kw OR 'wearable electronic device':ti,ab,kw OR 'wearable devices':ti,ab,kw OR 'device, wearable':ti,ab,kw OR 'wearable device':ti,ab,kw OR 'wearable technology':ti,ab,kw OR 'technology, wearable':ti,ab,kw OR 'wearable technologies':ti,ab,kw OR 'electronic skin':ti,ab,kw OR 'skin, electronic':ti,ab,kw OR 'wearable computer':ti,ab,kw OR 'computer, wearable':ti,ab,kw OR 'wearable computers':ti,ab,kw)  OR  ('behaviour therapy':ti,ab,kw OR 'behavioral therapy':ti,ab,kw OR 'cognitive therapy':ti,ab,kw OR 'cognitive behavioural therapy':ti,ab,kw OR 'cognitive behavioral therapy':ti,ab,kw OR 'CBT':ti,ab,kw OR 'ICBT':ti,ab,kw OR 'internet-based CBT':ti,ab,kw OR 'online CBT':ti,ab,kw OR 'web-based CBT':ti,ab,kw OR 'app-based CBT':ti,ab,kw OR 'computerized CBT':ti,ab,kw OR 'digital CBT':ti,ab,kw OR 'e-CBT':ti,ab,kw OR 'cognitive restructuring':ti,ab,kw OR 'cognitive remediation':ti,ab,kw)  #1 AND #2 (1925 2025-07-08) |
| Cochrane Library | #1  #2 | MeSH descriptor: [Urinary Bladder, Overactive] explode all trees OR overactive bladder:ti,ab OR overactive bladder syndrome:ti,ab OR bladder overactive:ti,ab OR overactive urinary bladder:ti,ab OR bladder overactivity:ti,ab OR OAB:ti,ab OR idiopathic overactive bladder:ti,ab  OR detrusor overactivity:ti,ab OR overactive detrusor:ti,ab OR overactive detrusor function:ti,ab OR detrusor function, overactive:ti,ab OR detrusor overactive:ti,ab OR detrusor overactivities:ti,ab OR detrusor instability:ti,ab OR idiopathic detrusor overactivity:ti,ab  OR MeSH descriptor: [Urinary Incontinence, Urge] explode all trees OR Urge Incontinence:ti,ab OR Incontinence, Urge:ti,ab OR UUI:ti,ab OR Urinary Reflex Incontinence:ti,ab OR Incontinence, Urinary Reflex:ti,ab OR Urinary Urge Incontinence:ti,ab OR urge-type urinary incontinence:ti,ab OR urgency incontinence:ti,ab OR Storage urinary symptoms:ti,ab  MeSH descriptor: [Telemedicine] explode all trees OR MeSH descriptor: [Therapy, Computer-Assisted] explode all trees OR MeSH descriptor: [Mobile Applications] explode all trees OR MeSH descriptor: [Internet] explode all trees OR MeSH descriptor: [Digital Health] explode all trees OR MeSH descriptor: [Wearable Electronic Devices] explode all trees OR MeSH descriptor: [Behavior Therapy] explode all trees OR  "ehealth":ti,ab OR "mhealth":ti,ab OR "electronic health":ti,ab OR "digital health":ti,ab OR "digital therapeutics":ti,ab OR "mobile health":ti,ab OR "digital medicine":ti,ab OR "telehealth":ti,ab OR "virtual care":ti,ab OR  "digital intervention":ti,ab OR "technology-based intervention":ti,ab OR "computer-assisted therapy":ti,ab OR "internet intervention":ti,ab OR "remote intervention":ti,ab OR "virtual intervention":ti,ab OR "online intervention":ti,ab OR "computerized intervention":ti,ab OR "mobile phone intervention":ti,ab OR "app-based intervention":ti,ab OR  "mobile app":ti,ab OR "mobile apps":ti,ab OR "app, mobile":ti,ab OR "apps, mobile":ti,ab OR "mobile application":ti,ab OR "smartphone app":ti,ab OR "app-based":ti,ab OR "smartphone":ti,ab OR  "health apps":ti,ab OR "wearable devices":ti,ab OR "smart devices":ti,ab OR  "web-based":ti,ab OR "internet-based":ti,ab OR "online":ti,ab OR "teleconferencing":ti,ab OR "teleconference":ti,ab OR "text messaging":ti,ab OR "sms intervention":ti,ab OR "remote monitoring":ti,ab OR  ("self-management":ti,ab AND ("digital":ti,ab OR "online":ti,ab)) OR  "wechat":ti,ab OR "portable electronic apps":ti,ab OR "portable electronic applications":ti,ab OR "electronic application, portable":ti,ab OR "portable electronic application":ti,ab OR "portable software apps":ti,ab OR "software":ti,ab OR "portable software applications":ti,ab OR "application, portable software":ti,ab OR "portable software application":ti,ab OR  "behaviour therapy":ti,ab OR "behavioral therapy":ti,ab OR "cognitive therapy":ti,ab OR "cognitive behavioural therapy":ti,ab OR "cognitive behavioral therapy":ti,ab OR "CBT":ti,ab OR "ICBT":ti,ab OR "internet-based CBT":ti,ab OR "online CBT":ti,ab OR "web-based CBT":ti,ab OR "app-based CBT":ti,ab OR "computerized CBT":ti,ab OR "digital CBT":ti,ab OR "e-CBT":ti,ab OR "cognitive restructuring":ti,ab OR "cognitive remediation":ti,ab  #1 AND #2 (423 2025-07-08) |
| CINAHL | #1  #2 | (MH "Urinary Bladder, Overactive") OR TI "overactive bladder" OR AB "overactive bladder" OR TI "overactive bladder syndrome" OR AB "overactive bladder syndrome" OR TI "bladder overactive" OR AB "bladder overactive" OR TI "overactive urinary bladder" OR AB "overactive urinary bladder" OR TI "bladder overactivity" OR AB "bladder overactivity" OR TI "OAB" OR AB "OAB" OR TI "idiopathic overactive bladder" OR AB "idiopathic overactive bladder"  OR TI "detrusor overactivity" OR AB "detrusor overactivity" OR TI "overactive detrusor" OR AB "overactive detrusor" OR TI "overactive detrusor function" OR AB "overactive detrusor function" OR TI "detrusor function, overactive" OR AB "detrusor function, overactive" OR TI "detrusor overactive" OR AB "detrusor overactive" OR TI "detrusor overactivities" OR AB "detrusor overactivities" OR TI "detrusor instability" OR AB "detrusor instability" OR TI "idiopathic detrusor overactivity" OR AB "idiopathic detrusor overactivity"  OR TI "Urge Incontinence" OR AB "Urge Incontinence" OR TI "Incontinence, Urge" OR AB "Incontinence, Urge" OR TI "UUI" OR AB "UUI" OR TI "Urinary Reflex Incontinence" OR AB "Urinary Reflex Incontinence" OR TI "Incontinence, Urinary Reflex" OR AB "Incontinence, Urinary Reflex" OR TI "Urinary Urge Incontinence" OR AB "Urinary Urge Incontinence" OR TI "urge-type urinary incontinence" OR AB "urge-type urinary incontinence" OR TI "urgency incontinence" OR AB "urgency incontinence" OR TI "Storage urinary symptoms" OR AB "Storage urinary symptoms"  (MH "Therapy, Computer Assisted+") OR (MH "Internet-Based Intervention+") OR (MH "Mobile Applications+") OR (MH "Cell Phone+") OR (MH "Telemedicine+") OR (MH "Wearable Electronic Devices") OR (MH "Behavior Therapy")  OR TI "eHealth" OR AB "eHealth"  OR TI "mHealth" OR AB "mHealth"  OR TI "Electronic Health" OR AB "Electronic Health"  OR TI "Digital Health" OR AB "Digital Health"  OR TI "Digital Therapeutics" OR AB "Digital Therapeutics"  OR TI "Mobile Health" OR AB "Mobile Health"  OR TI "Digital Medicine" OR AB "Digital Medicine"  OR TI "Telehealth" OR AB "Telehealth"  OR TI "Virtual Care" OR AB "Virtual Care"  OR TI "Digital Intervention" OR AB "Digital Intervention"  OR TI "Technology based Intervention" OR AB "Technology based Intervention"  OR TI "Computer assisted Therapy" OR AB "Computer assisted Therapy"  OR TI "Internet Intervention" OR AB "Internet Intervention"  OR TI "Remote Intervention" OR AB "Remote Intervention"  OR TI "Virtual Intervention" OR AB "Virtual Intervention"  OR TI "Online Intervention" OR AB "Online Intervention"  OR TI "Computerized Intervention" OR AB "Computerized Intervention"  OR TI "Mobile Phone Intervention" OR AB "Mobile Phone Intervention"  OR TI "App based Intervention" OR AB "App based Intervention"  OR TI "Mobile App" OR AB "Mobile App"  OR TI "Mobile Apps" OR AB "Mobile Apps"  OR TI "App Mobile" OR AB "App Mobile"  OR TI "Apps Mobile" OR AB "Apps Mobile"  OR TI "Mobile Application" OR AB "Mobile Application"  OR TI "Smartphone App" OR AB "Smartphone App"  OR TI "App based" OR AB "App based"  OR TI "Smartphone" OR AB "Smartphone"  OR TI "Health Apps" OR AB "Health Apps"  OR TI "Wearable Devices" OR AB "Wearable Devices"  OR TI "Smart Devices" OR AB "Smart Devices"  OR TI "Web based" OR AB "Web based"  OR TI "Internet based" OR AB "Internet based"  OR TI "Online" OR AB "Online"  OR TI "Teleconferencing" OR AB "Teleconferencing"  OR TI "Text Messaging" OR AB "Text Messaging"  OR TI "SMS Intervention" OR AB "SMS Intervention"  OR TI "Remote Monitoring" OR AB "Remote Monitoring"  OR ((TI "self-management" OR AB "self-management") AND (TI "digital" OR AB "digital" OR TI "online" OR AB "online"))  OR TI "wechat" OR AB "wechat"  OR TI "Portable Electronic Apps" OR AB "Portable Electronic Apps"  OR TI "Portable Electronic Applications" OR AB "Portable Electronic Applications"  OR TI "Electronic Application Portable" OR AB "Electronic Application Portable"  OR TI "Portable Electronic Application" OR AB "Portable Electronic Application"  OR TI "Portable Software Apps" OR AB "Portable Software Apps"  OR TI "software" OR AB "software"  OR TI "Portable Software Applications" OR AB "Portable Software Applications"  OR TI "Application Portable Software" OR AB "Application Portable Software"  OR TI "Portable Software Application" OR AB "Portable Software Application"  OR TI "Device Wearable Electronic" OR AB "Device Wearable Electronic"  OR TI "Electronic Device Wearable" OR AB "Electronic Device Wearable"  OR TI "Wearable Electronic Device" OR AB "Wearable Electronic Device"  OR TI "Wearable Devices" OR AB "Wearable Devices"  OR TI "Device Wearable" OR AB "Device Wearable"  OR TI "Wearable Device" OR AB "Wearable Device"  OR TI "Wearable Technology" OR AB "Wearable Technology"  OR TI "Technology Wearable" OR AB "Technology Wearable"  OR TI "Wearable Technologies" OR AB "Wearable Technologies"  OR TI "Electronic Skin" OR AB "Electronic Skin"  OR TI "Skin Electronic" OR AB "Skin Electronic"  OR TI "Wearable Computer" OR AB "Wearable Computer"  OR TI "Computer Wearable" OR AB "Computer Wearable"  OR TI "Wearable Computers" OR AB "Wearable Computers"  OR TI "behaviour therapy" OR AB "behaviour therapy"  OR TI "behavioral therapy" OR AB "behavioral therapy"  OR TI "cognitive therapy" OR AB "cognitive therapy"  OR TI "cognitive behavioural therapy" OR AB "cognitive behavioural therapy"  OR TI "cognitive behavioral therapy" OR AB "cognitive behavioral therapy"  OR TI "CBT" OR AB "CBT"  OR TI "ICBT" OR AB "ICBT"  OR TI "internet-based CBT" OR AB "internet-based CBT"  OR TI "online CBT" OR AB "online CBT"  OR TI "web-based CBT" OR AB "web-based CBT"  OR TI "app-based CBT" OR AB "app-based CBT"  OR TI "computerized CBT" OR AB "computerized CBT"  OR TI "digital CBT" OR AB "digital CBT"  OR TI "e-CBT" OR AB "e-CBT"  OR TI "cognitive restructuring" OR AB "cognitive restructuring"  OR TI "cognitive remediation" OR AB "cognitive remediation"  #1 AND #2(208 2025-07-08) |
| Web of Science | #1  #2 | TS=("overactive bladder" OR "overactive bladder syndrome" OR "bladder overactive" OR "overactive urinary bladder" OR "bladder overactivity" OR "OAB" OR "idiopathic overactive bladder" OR "detrusor overactivity" OR "overactive detrusor" OR "overactive detrusor function" OR "detrusor function, overactive" OR "detrusor overactive" OR "detrusor overactivities" OR "detrusor instability" OR "idiopathic detrusor overactivity" OR "Urge Incontinence" OR "Incontinence, Urge" OR "UUI" OR "Urinary Reflex Incontinence" OR "Incontinence, Urinary Reflex" OR "Urinary Urge Incontinence" OR "urge-type urinary incontinence" OR "urgency incontinence" OR "Storage urinary symptoms")  TS=("Telemedicine" OR "Mobile Applications" OR "Wearable Electronic Devices"  OR "eHealth" OR "mHealth" OR "Electronic Health" OR "Digital Health" OR "Digital Therapeutics" OR "Mobile Health" OR "Digital Medicine" OR "Telehealth" OR "Virtual Care"  OR "Digital Intervention" OR "Technology-based Intervention" OR "Computer-assisted Therapy" OR "Internet Intervention" OR "Remote Intervention" OR "Virtual Intervention" OR "Online Intervention" OR "Computerized Intervention" OR "Mobile Phone Intervention" OR "App-based Intervention"  OR "Mobile App" OR "Mobile Apps" OR "App, Mobile" OR "Apps, Mobile" OR "Mobile Application" OR "Smartphone App" OR "App-based" OR "Smartphone" OR "Health Apps" OR "Wearable Devices" OR "Smart Devices"  OR "Web-based" OR "Internet-based" OR "Online" OR "Teleconferencing" OR "Text Messaging" OR "SMS Intervention" OR "Remote Monitoring"  OR ("self-management" AND ("digital" OR "online"))  OR "wechat" OR "Portable Electronic Apps" OR "Portable Electronic Applications" OR "Electronic Application, Portable" OR "Portable Electronic Application" OR "Portable Software Apps" OR "software" OR "Portable Software Applications" OR "Application, Portable Software" OR "Portable Software Application"  OR "Device, Wearable Electronic" OR "Electronic Device, Wearable" OR "Wearable Electronic Device" OR "Wearable Devices" OR "Device, Wearable" OR "Wearable Device" OR "Wearable Technology" OR "Technology, Wearable" OR "Wearable Technologies" OR "Electronic Skin" OR "Skin, Electronic" OR "Wearable Computer" OR "Computer, Wearable" OR "Wearable Computers" OR "behaviour therapy" OR "behavioral therapy" OR "cognitive therapy" OR "cognitive behavioural therapy" OR "cognitive behavioral therapy" OR "CBT" OR "ICBT" OR "internet-based CBT" OR "online CBT" OR "web-based CBT" OR "app-based CBT" OR "computerized CBT" OR "digital CBT" OR "e-CBT" OR "cognitive restructuring" OR "cognitive remediation")  #1 AND #2 (638 2025-07-08) |
| 中国知网 | #1  #2 | 膀胱过度活动症 + 膀胱过度活动 + 膀胱活动过度 + OAB + 逼尿肌过度活动 + 逼尿肌高活动 + 急迫性尿失禁 + 尿急性尿失禁 + 尿急 + 尿频 + 夜间排尿 + 夜尿  微信 + 小程序 + app + eHealth + mHealth + 移动应用 + 手机应用 + 电子健康 + 电子医疗 + 数字健康 + 数字疗法 + 网络 + 互联网+医疗 + 网络干预 + 网络平台 + 智能 + 在线咨询 + 在线治疗 + 电话干预 + 远程医疗 + 远程干预 + 智能设备 + 可穿戴设备 + 认知行为疗法 + 认知行为治疗 + 行为认知 + 认知行为 + 行为疗法 + 行为治疗 + 认知疗法 + 心理干预 + 网络认知行为疗法 + 在线认知行为治疗 + CBT  #1 AND #2（457 2025-07-08） |
| 万方 | #1  #2 | 主题=("膀胱过度活动症" OR "膀胱过度活动" OR "膀胱活动过度" OR "OAB" OR "逼尿肌过度活动" OR "逼尿肌高活动" OR "急迫性尿失禁" OR "尿急性尿失禁" OR "尿急" OR "尿频" OR "夜间排尿" OR "夜尿")  主题=("微信" OR "小程序" OR "app" OR "eHealth" OR "mHealth" OR "移动应用" OR "手机应用" OR "电子健康" OR "电子医疗" OR "数字健康" OR "数字疗法" OR "网络" OR "互联网+医疗" OR "网络干预" OR "网络平台" OR "智能" OR "在线咨询" OR "在线治疗" OR "电话干预" OR "远程医疗" OR "远程干预" OR "智能设备" OR "可穿戴设备" OR "认知行为疗法" OR "认知行为治疗" OR "行为认知" OR "认知行为" OR "行为疗法" OR "行为治疗" OR "认知疗法" OR "心理干预" OR "网络认知行为疗法" OR "在线认知行为治疗" OR "CBT")  #1 AND #2（866 2025-07-08） |
| 中国生物医学文献服务系统(SinoMed) | #1  #2 | "膀胱过度活动症""[常用字段:智能] OR ""膀胱过度活动""[常用字段:智能] OR ""膀胱活动过度""[常用字段:智能] OR ""OAB""[常用字段:智能] OR ""逼尿肌过度活动""[常用字段:智能] OR ""逼尿肌高活动""[常用字段:智能] OR ""急迫性尿失禁""[常用字段:智能] OR ""尿急性尿失禁""[常用字段:智能] OR ""尿急""[常用字段:智能] OR ""尿频""[常用字段:智能] OR ""夜间排尿""[常用字段:智能] OR ""夜尿""[常用字段:智能]  "微信"[常用字段:智能] OR "小程序"[常用字段:智能] OR "app"[常用字段:智能] OR "eHealth"[常用字段:智能] OR "mHealth"[常用字段:智能] OR "移动应用"[常用字段:智能] OR "手机应用"[常用字段:智能] OR "电子健康"[常用字段:智能] OR "电子医疗"[常用字段:智能] OR "数字健康"[常用字段:智能] OR "数字疗法"[常用字段:智能] OR "网络"[常用字段:智能] OR "互联网OR医疗"[常用字段:智能] OR "网络干预"[常用字段:智能] OR "网络平台"[常用字段:智能] OR "智能"[常用字段:智能] OR "在线咨询"[常用字段:智能] OR "在线治疗"[常用字段:智能] OR "电话干预"[常用字段:智能] OR "远程医疗"[常用字段:智能] OR "远程干预"[常用字段:智能] OR "智能设备"[常用字段:智能] OR "可穿戴设备"[常用字段:智能] OR "认知行为疗法"[常用字段:智能] OR "认知行为治疗"[常用字段:智能] OR "行为认知"[常用字段:智能] OR "认知行为"[常用字段:智能] OR "行为疗法"[常用字段:智能] OR "行为治疗"[常用字段:智能] OR "认知疗法"[常用字段:智能] OR "心理干预"[常用字段:智能] OR "网络认知行为疗法"[常用字段:智能] OR "在线认知行为治疗"[常用字段:智能] OR "CBT"[常用字段:智能]  #1 AND #2 (306 2025-07-08) |
