# Supplementary material for: Effects of Digital Interventions on Symptom Improvement and Quality of Life in Patients With Overactive Bladder: Systematic Review and Meta-Analysis
Source: J Med Internet Res. 2026 Aug 10;28:e88971. doi: 10.2196/88971 (PMC13504251; doi:10.2196/88971)
Supplement: Supplementary file 3 [file jmir_v28i1e88971_app2.docx]

Multimedia Appendix 2. Characteristics of included studies

| Study/Country | Participants (IG/CG) | | Intervention | | Duration/Follow-up (Attrition rate) | Outcomes/Measures | Findings |
| --- | --- | --- | --- | --- | --- | --- | --- |
|  | Sample size | Mean age | IG | CG |  |  |  |
| R. Palmerola, et al [38] (2020)/America | 19/17 | Not reported | Content:telemedicine including a phone call follow up between office visits or to standard follow up No./length/frequency of session: at least 12 weeks  Format/delivery mode: face-to-face, telephone and email Setting: hospital and home. | standard follow up(consistent with the SUFU OAB CCP) | 12 weeks (12-week intervention)/ no information (36.11%) | QoL/OABq-SF | no significant differences were found in OABq-SF |
| Weinberger et al [16] (2025)  /America | 84/80 | 60.6/60.2 | Content: telemedicine via video visit, managed utilizing the SUFU OAB clinical care pathway No./length/frequency of session: baseline and up to six consecutive follow‐up visits Format/delivery mode: video Setting:in clinic or remotely(home). | traditional in-person clinic visits | 1 year(1-year intervention)/ no information(17.1%) | QoL/ OAB-q SF; PGI-S; PGI-I | Telemedicine was non-inferior to in-person visits |
| S. Mossack, et al [19] (2022)/America | 26/27 | 51/48 | Content:follow-up visits for OAB after initial behavioral modification and pharmacotherapy.  No./length/frequency of session:follow-up assessed at 30, 60, and 90 days.  Format/delivery mode:telemedicine (audiovisual).  Setting:remote(home). | in-person office visits. | 90 days/ 30-60- -90 days(9.4%) | OAB severity /UDI-6; IIQ-7  QoL/Patient Satisfaction  Follow-up rate | no significant differences were found |
| S. I. A. Alghitany, et al [17] (2024)/Egypt | 40/40 | 38.97/39.61 | Content: supervised group Pilates exercise program + Mediterranean diet.  No./length/frequency of session:3 times a week.  Format/delivery mode: videoconference.  Setting: home. | unsupervised home program of Pilates exercises + Mediterranean diet. | 12 weeks((12-week intervention) ) / at baseline and 12 weeks(6.25%) | OAB Severity/Patient Perception of Urgency Scale (PPIUS)  QoL/OAB-q | OAB Severity↓  QoL↑ |
| Feiwen Li, Yuanyuan Huang [39] (2020)/China | 42/43 | 57.38/60.37 | Content: health education based on PRECEDE-PROCEED model, including group classes, one-on-one training, and WeChat group support. No./length/frequency of session:3 group classes over 3 months, plus weekly phone/WeChat follow-ups.  Format/delivery mode: in-person classes (PPT), face-to-face training, WeChat, telephone.  Setting:hospital outpatient and remote. | routine health education (given informational pamphlet to read). | 3 months (3-month intervention)/ at baseline and 3 months(5.88%) | OAB severity/OABSS  QoL/OAB-q | OAB Severity↓  QoL↑ |
| S. Funada, et a [18] (2024)/Japan | 39/40 | 63.5/63.5 | Content: a multicomponent intervention including cognitive & behavioral skills (self-monitoring, education, CBT model, lifestyle modification, PFMT, bladder training, relapse prevention).  No./length/frequency of session: four 30-minute weekly sessions.  Format/delivery mode: individually, either face-to-face or via teleconferencing.  Setting:hospital or remote (home). | waiting list control group. (continued baseline treatment but received no new intervention during the study period). | 13 weeks (4-week intervention) /at weeks 0, 5, 9, and 13 (8.86%) | OAB severity /OABSS;frequency volume chart  QoL/OAB-q | QoL ↑  frequency volume chart↓  no significant difference in OABSS |
| A. D. Andrade, et al [37] (2015)/America | 25/22 | 62.41/60.68 | Content:  a 3-part online self-management program with a generic avatar coach and a personalized self-avatar peer mentor. Content included OAB symptoms, bladder diaries, lifestyle changes, PFMT, bladder training, etc.  No./length/frequency of session: part 1 at week 1; Parts 2 & 3 at week 6.  Format/delivery mode: online multimedia tutorials with embedded avatars.  Setting:home. | receiving the identical online program in a voice-only version without the visual avatars | 12 weeks(intervention at week 1 & 6) / at weeks 0, 1, 6, and 12 (12.77%) | OAB Severity/bladder Diary  QoL /OAB-q | OAB Severity ↓  QoL ↑ |

QoL (Quality of Life); OAB-q SF (Overactive Bladder Questionnaire Short Form); UDI-6 (Urogenital Distress Inventory-6); IIQ-7 / IIQ-SF (Incontinence Impact Questionnaire Short Form); PGI-S / PGI-I (Patient Global Impression of Severity / Improvement); PPIUS (Patient Perception of Intensity of Urgency Scale); OABSS (Overactive Bladder Symptom Score); IPSS QoL (International Prostate Symptom Score Quality of Life Question); EPIC (Expanded Prostate Cancer Index Composite); UCLA-PCI (University of California–Los Angeles Prostate Cancer Index); ICIQ-SF (International Consultation on Incontinence Questionnaire–Short Form); UISRP (Urinary Incontinence Scale of Radical Prostatectomy); EORTC-QLQ-C30 (European Organization for Research and Treatment of Cancer Quality of Life Questionnaire Core 30); SF-36 (Short Form Health Survey); PFMT (Pelvic Floor Muscle Training); CBT (Cognitive Behavioral Therapy)

**References**

[16] Weinberger JM, Gu C, Romeo H, Kwan L, Siavoshi M, Cabri J, Nitti VW. A randomized noninferiority trial for telemedicine in the follow-up of overactive bladder patients. Neurourol Urodyn 2025;44(7):1403-1411. doi:10.1002/nau.70123

[17] Alghitany SIA, Abd El-Monaem HA, Aziz MZ, Ibrahim NA, Guirguis SA. Telerehabilitation program impact on overactive bladder symptoms and metabolic health in obese women: a randomized controlled trial. Bull Rehabil Med 2024;23(4):47-54. doi:10.38025/2078-1962-2024-23-4-47-54

[18] Funada S, Luo Y, Uozumi R, Watanabe N, Goto T, Negoro H, Ueno K, Ichioka K, Segawa T, Akechi T, Ogawa O, Akamatsu S, Kobayashi T, Furukawa TA. Multicomponent intervention for overactive bladder in women: a randomized clinical trial. JAMA Netw Open 2024;7(3):e241784. doi:10.1001/jamanetworkopen.2024.1784

[19] Mossack S, Inoyatov I, Fonseca P, Du C, Lee E, Ruan H, Kim J. A randomized controlled trial comparing telemedicine versus in-person office visits for the follow-up of overactive bladder. Urogynecology 2022;28(12):819-824. doi:10.1097/SPV.0000000000001231

[37] Andrade AD, Anam R, Karanam C, Downey P, Ruiz JG. An overactive bladder online self-management program with embedded avatars: a randomized controlled trial of efficacy. Urology 2015;85(3):561-567. doi:10.1016/j.urology.2014.11.017

[38] Palmerola R, Escobar C, Sussman R, Brandon C, Smilen S, Pape D. Can telemedicine improve follow-up adherence and outcomes in patients with overactive bladder? SUFU 2020 Winter Meeting; 2020:239-240.

[39] Li F, Huang Y. Application study of health education based on the PRECEDE-PROCEED model in female patients with overactive bladder [in Chinese]. Pract Geriatr 2020;34(2):198-201. doi:10.3969/j.issn.1003-9198.2020.02.026
