# Supplementary material for: Effects of Digital Interventions on Symptom Improvement and Quality of Life in Patients With Overactive Bladder: Systematic Review and Meta-Analysis
Source: J Med Internet Res. 2026 Aug 10;28:e88971. doi: 10.2196/88971 (PMC13504251; doi:10.2196/88971)
Supplement: Supplementary file 4 [file jmir_v28i1e88971_app3.docx]

Multimedia Appendix 3. Summary of Findings

| Certainty assessment | | | | | | | № of patients | | Effect | | Certainty | Importance |
| --- | --- | --- | --- | --- | --- | --- | --- | --- | --- | --- | --- | --- |
| № of studies | Study design | Risk of bias | Inconsistency | Indirectness | Imprecision | Other considerations | Intervention | Control | Relative (95% CI) | Absolute (95% CI) |  |  |
| Objective symptom indicators | | | | | | | | | | | | |
| 24-hour urinary frequency | randomised trials | seriousa | not seriousb | not serious | not serious | none | 56 | 57 | - | MD 2.55 lower (4.22 lower to 0.89 lower) | ⨁⨁⨁◯ Moderatea,b | critical |
| Objective symptom indicators | | | | | | | | | | | | |
| Night-time urination | randomised trials | seriousa | seriousc | not serious | seriousd | none | 56 | 57 | - | MD 0.84 lower (1.91 lower to 0.22 higher) | ⨁◯◯◯ Very lowa,c,d | critical |
| Objective symptom indicators | | | | | | | | | | | | |
| Urge incontinence episodes | randomised trials | seriousa | not serious | not serious | not serious | none | 56 | 57 | - | MD 2.07 lower (2.72 lower to 1.42 lower) | ⨁⨁⨁◯ Moderatea | critical |
| OAB Symptom-Specific Questionnaire score | | | | | | | | | | | | |
| Subjective Symptom Scores | randomised trials | seriouse | very seriousf | not serious | not serious | none | 213 | 202 | - | SMD 1.07 SD lower (2.18 lower to 0.05 higher) | ⨁◯◯◯ Very lowe,f | critical |
| OAB-specific quality of life score | | | | | | | | | | | | |
| Quality of Life (QoL) | randomised trials | seriousg | serioush | not serious | not serious | none | 93 | 95 | - | SMD 1.47 SD higher (0.11 higher to 2.84 higher) | ⨁⨁◯◯ Lowg,h | critical |
| attrition rates | | | | | | | | | | | | |
| Attrition rates (Dropout) | randomised trials | seriousi | not serious | not serious | seriousj | none | 32/275 (11.6%) | 32/269 (11.9%) | RR 0.92 (0.59 to 1.45) | 10 fewer per 1,000 (from 49 fewer to 54 more) | ⨁⨁◯◯ Lowi,j | important |

a. Downgraded one level for serious risk of bias. The analysis is based on two RCTs. One study (Andrade et al [37) was rated at an overall high risk of bias, particularly due to risks in the domains of 'deviations from intended interventions' and 'missing outcome data'. The other study had some concerns. The inclusion of a study at high risk of bias seriously limits our confidence in the effect estimate.

b. Although the statistical heterogeneity is substantial (I² = 73%), we did not downgrade for inconsistency because the analysis is based on only two studies, and their results are consistent in the direction of effect. Both studies favored the digital intervention.

c. Downgraded one level for serious inconsistency. The analysis showed substantial statistical heterogeneity (I² = 90%), and the results of the two studies were conflicting in direction and magnitude.

d. The pooled 95% confidence interval [-1.91, 0.22] clearly crosses the line of no effect (0).

e. Downgraded one level for serious risk of bias. The analysis is based on five RCTs. According to the original meta-analysis (Figure 2), four out of the five studies were rated at high risk of bias or had some concerns.

f. Downgraded two levels for very serious inconsistency. The analysis showed considerable statistical heterogeneity $I^{2}=96$.

g. Downgraded one level for serious risk of bias. The analysis is based on three RCTs. According to the original meta-analysis (Figure 2), at least two of the three studies (Andrade et al. [37] and Funada et al [18]) were rated as having a high risk of bias or raised some concerns.

h. Downgraded one level for serious inconsistency. The analysis showed substantial statistical heterogeneity (I² = 94%),

i. Downgraded one level for serious risk of bias. The analysis is based on seven RCTs. According to the original meta-analysis (Figure 2), the majority of the included studies were rated as being at high risk of bias or had some concerns.

j. Downgraded one level for serious imprecision. The 95% confidence interval for the risk ratio [0.59, 1.45] is wide and includes both clinically significant benefit and harm, crossing the line of no effect.

**References**

[18] Funada S, Luo Y, Uozumi R, Watanabe N, Goto T, Negoro H, Ueno K, Ichioka K, Segawa T, Akechi T, Ogawa O, Akamatsu S, Kobayashi T, Furukawa TA. Multicomponent intervention for overactive bladder in women: a randomized clinical trial. JAMA Netw Open 2024;7(3):e241784. doi:10.1001/jamanetworkopen.2024.1784

[37] Andrade AD, Anam R, Karanam C, Downey P, Ruiz JG. An overactive bladder online self-management program with embedded avatars: a randomized controlled trial of efficacy. Urology 2015;85(3):561-567. doi:10.1016/j.urology.2014.11.017
